# COVID-19 and Economic Growth: Does Good Government Performance Pay Off?

**DOI:** 10.1007/s10272-020-0906-0

**Published:** 2020-07-28

**Authors:** Michael König, Adalbert Winkler

**Affiliations:** International and Development Finance, Frankfurt School of Finance and Management, Adickesallee 32–34, 60322 Frankfurt a. M., Germany

## Abstract

The coronavirus pandemic led to substantial revisions of 2020 GDP growth projections. We analyse whether and to what extent the quality of government policies in handling the health aspects of the crisis influence cross-country differences in the economic impact of the pandemic as projected by the OECD, the IMF and the World Bank. We measure policy quality by a recently published Economist Intelligence Unit index and a COVID-19 Misery index combining the stringency of government-imposed distancing measures with the COVID-19 fatality rate. Moreover, we control for international spillovers captured by trade openness and export exposure to tourism. Results for most specifications show that good government performance pays off as the respective countries record less severe revisions of growth forecasts. Only in a few cases, our findings suggest that the pandemic’s global effect might be so strong that actions by individual governments do not affect cross-country differences of growth revisions. Finally, there is broad evidence supporting the view that a country’s exposure to the global economy influences its growth outlook relative to other countries.

The COVID-19 pandemic is a global phenomenon. However, different countries are affected differently in terms of output losses. We test whether cross-country differences in economic impact reflect the degree to which countries successfully manage the pandemic. Our analysis is motivated by an index recently published by the Economist Intelligence Unit (EIU, 2020) designed to capture how well 21 Organisation for Economic Co-operation and Development (OECD) countries have responded to the coronavirus. We add to this index a COVID-19 Misery (CM) index which allows us to expand the analysis to all OECD countries and to several additional countries. In its baseline version, the CM index represents the product of the number of COVID-19 deaths per one million inhabitants with the government stringency index reflecting the degree of rigidity with which governments impose mandatory forms of social distancing (Hale et al., 2020).

**Michael König**, Frankfurt School of Finance & Management, FS UNEP Collaborating Centre for Climate and Sustainable Energy Finance, Germany.

We make use of both indices and test whether they significantly explain cross-country differences of revisions in GDP growth projections for 2020 recently published by the OCED (2020), the International Monetary Fund (IMF) (2020a) and the World Bank (2020) in June 2020. We account for international transmission channels (Lane and Milesi-Ferretti, 2011), as they are likely to play a major role in explaining differences in relative economic performance across countries. Even if there had been one country unaffected by the pandemic itself, i.e. recording zero infections and hence no increase in social distancing, economic activity would have likely declined due to its integration into the global economy (Rathke et al., 2020). We capture the level of integration by the degree of trade openness and the exposure of export revenues to tourism (Gössling et al., 2020). Finally, we include pre-crisis GDP growth to control for convergence effects.

The findings show that countries whose governments are handling the pandemic more successfully record revisions of growth forecasts that are less severe than countries with poorly performing governments. This result stands up to several robustness checks. In only a few specifications do the results suggest that the pandemic's global effect might be so strong that individual government actions do not affect cross-country differences in growth revisions. Finally, international spillovers matter, as we often find that a country's exposure to the global economy significantly influences its relative vulnerability to the pandemic in terms of projected growth. We conclude that despite the global character of the pandemic and the economic crisis it has triggered, the way individual governments handle the crisis will impact economic outcomes across countries.

### COVID-19 policies

There is no country unaffected by COVID-19. Accordingly, all governments have responded to the pandemic, mainly by imposing some form of mandatory social distancing. However, the effectiveness of the measures taken varies, as governments might have responded too late and too timidly, or too early and too harshly. This is not surprising as governments faced several challenges when responding to the coronavirus crisis. First, they did not know how fast the virus was spreading, how many people became infected and how high the fatality rate would turn out to be. Second, they did not know to what extent voluntary measures of social distancing would suffice to contain the pandemic in such a way that people would judge the associated fatality rate as acceptable. Thus, they were hesitant in applying restrictions even if government-imposed measures might have more efficiently achieved the level of social distancing needed to address the crisis (Eichenbaum et al., 2020).

Third, the government response was influenced by many country-specific factors (Deb et al., 2020; Pepinsky, 2020; Wharton School, 2020). For example, the fragility of a country and its political cohesion likely played a role: Governments of fragile states and countries characterised by a high degree of polarisation might have been less able to rely to a large extent on moral suasion in reaching the desired overall level of social distancing. In addition, governments' assessments of the danger the coronavirus presents have varied. For example, views expressed by the Brazilian President and the German Chancellor on COVID-19 suggest that the response to the crisis in Brazil and Germany was different. Related to this, governments' views on the political fallout of mandatory measures, i.e. whether they will consolidate or undermine the government in power, likely influenced how hesitant or forceful they responded by imposing mandatory measures. In addition, government policies presumably varied with the quality of the health sector: Governments of countries with better health systems have more leeway to contemplate mandatory measures given system abilities to cope with a larger number of infected people. Finally, the fatality rate and social distancing measures are endogenous to each other. A higher number of deaths raises risk aversion and hence leads to stronger voluntary and government-imposed measures of social distancing, while stronger voluntary and government responses are likely reflected by a lower number of deaths.

The EIU (2020) aims at addressing these complexities by designing an index expressing the quality of the government response to the pandemic. The EIU index accounts for three 'quality of response' criteria (number of tests, provision of non-COVID-19 healthcare and the number of above-average excess deaths) and three pre-existing risk factors (share of older population, obesity prevalence and number of international arrivals). The index is available for 21 OECD countries (Table 1). It indicates that New Zealand, Austria, Germany, Australia, Denmark, Iceland, Israel and Norway have performed best in managing the pandemic by acting swiftly and early, largely avoiding very tight lockdowns and running substantial tracking and testing programmes. As a result, these countries record a relatively low fatality rate, even though rates are quite heterogeneous within the group, and have been able to provide healthcare services to non-COVID-19 patients despite a significant share of the population above 65. By contrast, Spain, the UK, Italy and Belgium score worst either due to bad luck because they were the first countries in Europe to be hit by the pandemic or due to policies that were too slow and inconsistent. Portugal, Chile, France and the US fall in the middle, followed by Switzerland, Japan, South Korea, Sweden and the Netherlands.

Given the limited number of countries for which the EIU index is available, we apply the Corona Misery index for larger samples. As the name indicates, it uses the logic of the macroeconomic misery index developed by Arthur Okun (Cohen et al., 2014) to the pandemic. However, while Okun's index represents the sum of two unfavourable macroeconomic outcomes, inflation and unemployment, the CM index is the product of two unfavourable pandemic outcomes, namely the number of deaths per one million inhabitants and the stringency of the government-imposed measures (Hale et al., 2020). Correlation analysis reveals a significant negative correlation between the CM index and the EIU index (-0.786, $p<0.05$).

Despite the negative correlation, the CM index suggests a different ranking of the 21 countries in terms of the quality of the government response to the health crisis than the EIU index (see Table 1 and Figure 1). This is because the number of deaths per one million inhabitants drives the CM index ranking. Accordingly, countries showing a low fatality rate (such as South Korea and Japan) perform better, and countries with a relatively high fatality rate (such as Austria, Germany, the US and France) perform worse relative to the EIU index. By contrast, Sweden's ranking is almost unchanged as the negative impact of the high fatality rate for the CM index is also reflected in factors accounted for by the EIU index. Thus, Sweden's substantially more liberal policy approach (Born et al., 2020; Winkler, 2020) does not pay off in either ranking.

Table 1
**EIU and CM indices for country ranking**

| Country | EIU | Rank | CM (ln) | Rank | Stringency | Rank | Deaths per million | Rank | Start stringency | Delay |
|---|---|---|---|---|---|---|---|---|---|---|
| New Zealand | 3.67 | 1 | 5.27 | 2 | 41.86 | 11 | 4.64 | 2 | 21 | -37 |
| Austria | 3.56 | 2 | 8.06 | 8 | 40.44 | 9 | 77.94 | 8 | 23 | -4 |
| Germany | 3.56 | 3 | 8.37 | 9 | 40.32 | 8 | 107.39 | 10 | 54 | -2 |
| Australia | 3.44 | 4 | 5.12 | 1 | 40.11 | 6 | 4.14 | 1 | 57 | 0 |
| Denmark | 3.44 | 5 | 8.38 | 10 | 41.72 | 10 | 104.83 | 9 | 24 | 0 |
| Iceland | 3.44 | 6 | 6.85 | 5 | 31.75 | 3 | 29.7 | 5 | 22 | -37 |
| Israel | 3.44 | 7 | 7.55 | 7 | 51.55 | 19 | 36.75 | 6 | 26 | -26 |
| Norway | 3.44 | 8 | 7.48 | 6 | 38.06 | 4 | 46.65 | 7 | 30 | -27 |
| Portugal | 3.22 | 9 | 8.84 | 11 | 46.05 | 17 | 150.45 | 11 | 25 | -37 |
| Chile | 3.11 | 10 | 9.21 | 13 | 39.53 | 5 | 252.6 | 13 | 32 | 12 |
| France | 3.11 | 11 | 10.08 | 17 | 51.94 | 20 | 457.47 | 16 | 73 | 10 |
| USA | 3.11 | 12 | 9.70 | 16 | 43.84 | 15 | 372.91 | 15 | 22 | -2 |
| Switzerland | 2.89 | 13 | 8.98 | 12 | 40.30 | 7 | 197.17 | 12 | 55 | -1 |
| Japan | 2.89 | 14 | 5.44 | 3 | 30.80 | 2 | 7.61 | 4 | 6 | -8 |
| South Korea | 2.78 | 15 | 5.48 | 4 | 43.28 | 14 | 5.51 | 3 | 30 | 11 |
| Sweden | 2.56 | 16 | 9.51 | 14 | 25.73 | 1 | 522.38 | 17 | 68 | 37 |
| Netherlands | 2.44 | 17 | 9.63 | 15 | 42.54 | 13 | 357.39 | 14 | 65 | 7 |
| Spain | 2.22 | 18 | 10.28 | 19 | 47.55 | 18 | 615.77 | 19 | 30 | -1 |
| UK | 2.22 | 19 | 10.21 | 18 | 42.26 | 12 | 641.64 | 20 | 32 | 2 |
| Italy | 2.22 | 20 | 10.35 | 20 | 54.62 | 21 | 571.94 | 18 | 22 | -8 |
| Belgium | 2.11 | 21 | 10.56 | 21 | 45.71 | 16 | 846.7 | 21 | 27 | -7 |

Notes: The presented *CM* index is displayed in logarithmic form ln(x+1) and represents the product of the total number of COVID-19 *Deaths per million* inhabitants as of 28 June and the *Stringency* mean value between 1 January and 28 June 2020. *Start stringency* accounts for the number of days from 1 January 2020 without mandatory measures (e.g. 14 March 2020 = 74). *Delay* represents the period in days between starting mandatory measures (*Start stringency*) and the first registered COVID-19 infection.

Sources: Economist Intelligence Unit (2020), How well have OECD countries responded to the coronavirus crisis? – A report by The Economist Intelligence Unit; T. Hale, S. Webster, A. Petherick, T. Phillips and B. Kira (2020), Variation in government responses to COVID-19, Oxford COVID-19 Government Response Tracker, *BSG Working Paper Series*, BSG-WP-2020/032, www.bsg.ox.ac.uk/covidtracker (27 June 2020); authors' compilation.

## The COVID-19 policy response and economic growth

The COVID-19 pandemic has had a severe negative impact on economic activity. This is reflected in dramatic revisions of GDP growth forecasts. The IMF (2020b) growth forecasts for 2020 declined for all countries compared to those published in late autumn 2019, i.e. before the pandemic. Still, cross-country differences within projections are substantial. For example, the most recent IMF forecast (2020a) records a revision for GDP growth in 2020 of -2.75 percentage points for Pakistan, while that of Spain amounts to -14.65 percentage points (Table 2).

Moreover, on average, revisions have become more severe the more time elapses and the more samples focus on advanced economies. Thus, correlations of projected revisions are far from perfect. Indeed, for countries covered in the recent OECD and IMF projections, the correlation coefficient of growth revisions compared to late 2019 is just +0.39 and fails to be significant at a 10% level. The respective projections also differ markedly regarding their country coverage. While the OECD sample covers all of its member countries, the IMF World Economic Outlook (WEO) update released in June 2020 provides projections for 30 countries only. The World Bank just released new projections for 46 countries, but as Oxford University does not include many of them in the stringency index sample, our analysis of these projections covers only 29 countries.

Figure 1
**EIU and CM indices for 21 countries**

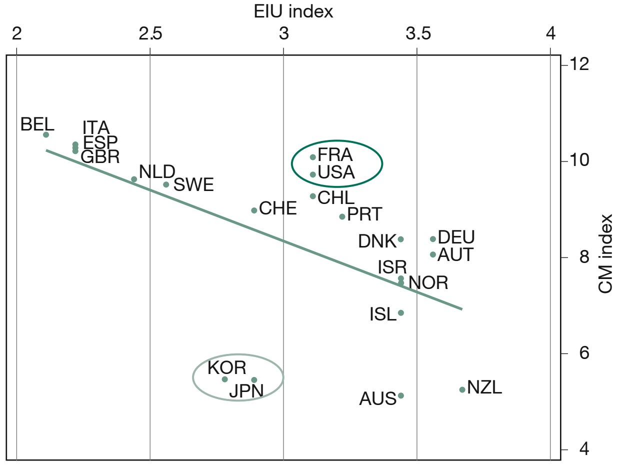

Sources: Economist Intelligence Unit (2020), How well have OECD countries responded to the coronavirus crisis? – A report by The Economist Intelligence Unit; T. Hale, S. Webster, A. Petherick, T. Phillips and B. Kira (2020), Variation in government responses to COVID-19, Oxford COVID-19 Government Response Tracker, *BSG Working Paper Series*, BSG-WP-2020/032, www.bsg.ox.ac.uk/covidtracker (27 June 2020); authors' compilation.

Finally, the composition of countries varies substantially. While OECD member countries include larger and smaller advanced economies plus several key emerging markets, the IMF WEO update focuses on the larger advanced and emerging markets only. The World Bank sample, by contrast, is mainly composed of developing countries.

Against this background, we test whether the quality of the government response as captured by the EIU and the CM indices significantly explains cross-country heterogeneity in terms of growth revisions associated with the pandemic, i.e. we test whether good government performance in handling the pandemic is likely to pay off. We apply a framework developed by Lane and Milesi-Ferretti (2011) and Blanchard and Leigh (2013) for testing the impact of various factors and policies on the depth of the recession countries experienced in the global financial crisis. Our choice reflects the finding that the global financial crisis is arguably the closest point of reference in post-World War II history with regard to growth outcomes we are likely to observe in 2020.

Concretely, we run the following regression (Equation 1):

$$(1) \quad \Delta y_{i2020} = \alpha + \beta_1 \text{*POLCOVID}_i + \beta_2 \text{*INT}_i + \beta_3 \text{*Zi} + \varepsilon_i$$

where $\Delta y_{i2020}$ is the change in the 2020 GDP growth projection of country *i* from the autumn 2019 to the sum-

Table 2
**Descriptive statistics**

| | Mean | Median | SD | Minimum | Maximum | Countries |
|---|---|---|---|---|---|---|
| Growth revisions | | | | | | |
| OECD | -9.419 | -9.60 | 1.89 | -12.77 | -3.53 | 47 |
| IMF | -8.517 | -8.62 | 2.84 | -14.65 | -2.75 | 29 |
| World Bank | -5.563 | -5.30 | 2.42 | -14.60 | -1.00 | 46 |
| COVID-19 responses | | | | | | |
| CM index | 7.859 | 7.80 | 1.57 | 4.69 | 10.56 | 47 |
| EIU index | 2.994 | 3.11 | 0.52 | 2.11 | 3.67 | 21 |
| Controls | | | | | | |
| Openness | 95.003 | 77.85 | 63.47 | 27.54 | 387.10 | 47 |
| Tourism | 8.110 | 5.43 | 6.24 | 1.52 | 26.38 | 47 |
| Trend growth | 2.694 | 2.48 | 1.51 | -0.62 | 6.73 | 47 |
| Instruments | | | | | | |
| Delay | -14.745 | -5.00 | 21.99 | -62.00 | 37.00 | 47 |
| Start stringency | 33.766 | 27.00 | 18.95 | 0.00 | 73.00 | 47 |
| State fragility | 40.007 | 38.26 | 19.97 | 14.63 | 79.15 | 47 |

Notes: The observations for *Controls* and *Instruments* are based on the OECD sample (n=47). Values for *Growth revisions* represent the respective full samples. CM index is the logarithm value ln(x+1) of the product of deaths per million inhabitants as of 28 June and the *Stringency* mean value of between 1 January and 28 June 2020. *Openness* is the sum of exports and imports divided by GDP in 2018. *Tourism* is the share of international tourism receipts in total exports in 2018. *Trend growth* is the mean average GDP growth rate over the period 2015-2019. *Start stringency* accounts for the number of days from 1 January 2020 without mandatory measures (e.g. 14 March 2020 = 74). *Delay* represents the period in days between starting mandatory measures (*Start stringency*) and the first registered COVID-19 infection. *State fragility* is measured by the Fund for Peace in 2018.

Source: Authors' own calculation.

Table 3
**GDP growth revisions and the quality of government response – baseline**

| Variables | (1) OECD | (2) OECD | (3) OECD | (4) IMF | (5) World Bank |
|---|---|---|---|---|---|
| EIU index | 2.127***<br>(0.688) | | | | |
| CM index | | -0.898***<br>(0.212) | -0.614***<br>(0.219) | -0.820***<br>(0.262) | -0.523***<br>(0.170) |
| Openness | -0.003<br>(0.010) | 0.005<br>(0.010) | -0.005<br>(0.005) | -0.019<br>(0.016) | -0.005<br>(0.015) |
| Tourism | -0.276**<br>(0.101) | -0.245***<br>(0.075) | -0.095**<br>(0.036) | -0.076<br>(0.129) | 0.029<br>(0.030) |
| Trend growth | 0.661<br>(0.911) | 0.300<br>(0.500) | -0.364***<br>(0.120) | 0.234<br>(0.319) | -0.031<br>(0.146) |
| Constant | -14.377***<br>(2.501) | -0.590<br>(1.453) | -2.347<br>(1.988) | -1.442<br>(2.276) | -2.674*<br>(1.506) |
| Sample | EIU countries | | Full samples | | |
| Number of countries | 21 | 21 | 47 | 28 | 29 |
| Adj. $R^2$ | 0.367 | 0.609 | 0.255 | 0.324 | 0.301 |
| F-Statistic | 11.996 | 14.201 | 4.992 | 4.126 | 5.422 |

Notes: Dependent variables are the changes in OECD (1), (2), (3), IMF (4) and World Bank (5) GDP growth revisions for 2020 from autumn 2019 to summer 2020. *,**,*** denote significance at 10%, 5%, and 1% levels, respectively. OLS estimations. Robust standard errors in parentheses. *CM index* is the logarithm value ln(x+1) of the Oxford University stringency index mean value from 1 January to 28 June 2020 times the number of COVID-19 deaths per million inhabitants on 28 June 2020. *Openness* is the sum of exports and imports divided by GDP in 2018. *Tourism* is the share of international tourism receipts in total exports in 2018. *Trend growth* is the mean average GDP growth rate over the period 2015-2019.

Source: Authors' own calculation.

mer 2020 projection made by the OECD (2020), the IMF (2020a) and the World Bank (2020). $POLCOVID_i$ either represents the EIU or the CM index, i.e. the quality of government policies in handling the crisis. The CM index is calculated by multiplying the mean of the Oxford University stringency index for the period 1 January to 28 June 2020 with the number of COVID-19 deaths per million inhabitants as of 28 June 2020. Given the skewed nature of the cross-sectional distribution of the CM index, the variable is employed in log form. $INT_i$ is a vector representing 2018 values for trade openness, defined as the sum of exports and imports divided by GDP, and the share of tourism receipts in total exports of countries. Finally, we follow Lane and Milesi-Ferretti (2011) and account for a general control variable $Z_i$. However, as the number of countries covered by the EIU index and by the various recently published projections is limited, we opt for a parsimonious approach and only account for average GDP growth over the five years preceding 2020 to capture convergence effects, i.e. we refrain from including population density and GDP per capita (König and Winkler, 2020).

## Results and discussion

The baseline has five specifications of Equation 1 (Table 3). We start with two specifications (1-2) based on the 21 countries for which the EIU index is available and test for the significance of the EIU index and the CM index. We find in both specifications that the quality of the government response matters: countries with a higher EIU index and a lower CM index record less severe revisions of growth projections for 2020. Moreover, cross-country heterogeneity in terms of tourism exposure significantly influences the results. Countries depending more on tourism revenues show significantly greater negative growth revisions than countries where tourism plays a less pronounced role in total exports. By contrast, differences in trade openness have no influence on growth revisions.[1]

Specifications 3-5 capture policy quality by the CM index only, as the respective samples are either too differ-

1 This is also the result for most specifications in the Lane and Milesi-Ferretti (2011) analysis of the global financial crisis.

Table 4

**GDP growth revisions and the quality of government response in the fourth quartile**

| | (1) OECD | (2) OECD | (3) OECD | (4) IMF | (5) World Bank |
|---|---|---|---|---|---|
| Lowest EIU index | -2.686*** (0.856) | | | | |
| Lowest CM index | | 2.283** (0.923) | 1.463 (0.889) | 2.076** (0.900) | 1.376 (1.572) |
| Openness | 0.001 (0.009) | -0.002 (0.011) | -0.006 (0.004) | 0.023 (0.013) | -0.005 (0.019) |
| Tourism | -0.265*** (0.080) | -0.276** (0.097) | -0.094** (0.045) | -0.070 (0.132) | 0.032 (0.045) |
| Trend growth | 0.791 (0.748) | 0.783 (0.585) | -0.268* (0.154) | 0.495 (0.307) | 0.058 (0.175) |
| Constant | -7.992*** (1.482) | -9.006*** (1.422) | -7.756*** (0.726) | -8.505*** (1.068) | -6.280*** (1.442) |
| Sample | EIU countries | | Full samples | | |
| Number of countries | 21 | 21 | 47 | 28 | 29 |
| Adj. $R^2$ | 0.440 | 0.358 | 0.126 | 0.153 | 0.058 |
| F-Statistic | 7.456 | 3.042 | 2.438 | 4.245 | 2.858 |

Notes: *Lowest EIU index and Lowest CM index* (fourth quartiles) represent dummy variables which take the value 1 if the country is in the first quartile of the EIU index (high index countries perform better than low index countries) and the CM index (low index countries perform better than high index countries) distribution.

Source: Authors' own calculation.

ent (IMF, 2020; World Bank, 2020) or too large (OECD, 2020) in terms of country coverage to run an analysis with the EIU index as the policy variable of interest.[2] Results confirm the explanatory power of the CM index for all samples. However, tourism loses significance in specifications for the IMF and World Bank samples. This is not surprising as both are widely based on large advanced and/or emerging market countries where exposure to tourism is not a major characteristic of export revenues.

We employ several robustness checks. First, we test for the economic impact when countries belong to the first quartile of either index (Table 4). Results confirm the baseline for EIU and IMF sample countries. However, OECD and World Bank sample countries with governments grouped in the first quartile do not show significantly different growth revisions due their government crisis management. Indeed, the World Bank sample estimation suggests that none of the variables we account for has explanatory power for cross-country differences in projected GDP growth revisions.

2 The overlap between the IMF (World Bank) and EIU country sample is restricted to ten (one) countries, only.

Secondly, we test the robustness of our results by changing the way the CM index is calculated. Concretely, we

Table 5

**Ranking of GDP growth revisions and the quality of government response**

| | (1) OECD | (2) OECD | (3) IMF | (4) World Bank |
|---|---|---|---|---|
| Misery ranking | -0.143*** (0.027) | -0.043*** (0.013) | -0.123*** (0.034) | -0.061 (0.040) |
| Openness | -0.002 0.009) | -0.010** (0.005) | -0.029* 0.014) | -0.008 (0.018) |
| Tourism | -0.237** 0.096) | -0.097** (0.041) | -0.089 (0.116) | 0.035 (0.031) |
| Trend growth | 0.444 (0.604) | -0.203* (0.112) | 0.503* (0.290) | 0.065 (0.153) |
| Constant | -4.818*** (0.924) | -5.107*** (1.047) | -3.908** (1.447) | -3.933* (2.243) |
| Sample | EIU countries | Full samples | | |
| Number of countries | 21 | 47 | 28 | 29 |
| Adj. $R^2$ | 0.589 | 0.221 | 0.297 | 0.133 |
| F-Statistic | 11.412 | 4.765 | 5.055 | 2.963 |

Notes: Misery ranking is the sum of the country ranking values for the stringency index ranking and the deaths per million ranking. A lower Misery ranking indicates a better performance.

Source: Authors' own calculation.

Table 6
**Instrumental variables in GDP growth revisions and the quality of government response**

| Variables | (1) OECD | (2) OECD | (3) OECD | (4) IMF | (5) World Bank |
|---|---|---|---|---|---|
| | A1: Second Stage | | A2: Second Stage | | |
| CM index | | -0.570**<br>(0.247) | 0.472<br>(0.488) | 0.437<br>(0.624) | -1.279**<br>(0.558) |
| EIU index | 1.429*<br>(0.737) | | | | |
| Openness | -0.006<br>(0.010) | -0.001<br>(0.008) | -0.010**<br>(0.005) | -0.022*<br>(0.013) | -0.012<br>(0.017) |
| Tourism | -0.273***<br>(0.087) | -0.253***<br>(0.071) | -0.050<br>(0.053) | -0.069<br>(0.123) | -0.006<br>(0.043) |
| Trend growth | 0.795<br>(0.775) | 0.581<br>(0.497) | 0.092<br>(0.269) | 0.704*<br>(0.397) | -0.196<br>(0.201) |
| Constant | -12.405***<br>(2.779) | -3.431<br>(2.171) | -12.009***<br>(4.418) | -11.873**<br>(5.312) | 2.938<br>(4.494) |
| Adj. $R^2$ | 0.344 | 0.548 | . | . | . |
| F-Statistic | 6.474 | 7.157 | 5.882 | 3.059 | 2.246 |
| | B1: First Stage | | B2: First Stage | | |
| Start stringency | 0.011*<br>(0.006) | 0.025<br>(0.023) | 0.010<br>(0.018) | 0.033<br>(0.031) | 0.018<br>(0.030) |
| Delay | -0.022***<br>(0.006) | 0.037<br>(0.024) | 0.014<br>(0.016) | -0.022<br>(0.019) | 0.013<br>(0.019) |
| State fragility | -0.018**<br>(0.007) | 0.075***<br>(0.021) | 0.006<br>(0.010) | -0.032<br>(0.025) | -0.049<br>(0.031) |
| Constant | 3.572***<br>(0.461) | 4.805**<br>(2.046) | 8.464***<br>(1.050) | 8.959***<br>(1.751) | 11.110***<br>2.458) |
| Sample | EIU countries | | Full sample | | |
| Number of countries | 21 | 21 | 47 | 28 | 29 |
| Adj. $R^2$ | 0.394 | 0.390 | 0.170 | 0.045 | 0.156 |
| F-Statistic for weak identification | 5.729 | 5.310 | 2.049 | 0.684 | 0.987 |
| Sargan stat. (p-value) | 0.202 | 0.089 | 0.684 | 0.790 | 0.888 |
| Wooldridge (p-value) | 0.353 | 0.102 | 0.050 | 0.003 | 0.035 |

Notes: Dependent variables are the changes in OECD (1), (2), (3), IMF (4), and World Bank (5) GDP growth revisions for 2020 from autumn 2019 to summer 2020. *,**,*** denote significance at 10%, 5%, and 1% levels, respectively. OLS estimations. Robust standard errors in parentheses. *CM index* is the logarithm value ln(x+1) of the Oxford University stringency index mean value from 1 January to 28 June 2020 times the number of COVID-19 deaths per million inhabitants on 28 June 2020. *Openness* is the sum of exports and imports divided by GDP in 2018. *Tourism* is the share of international tourism receipts in total exports in 2018. *Trend growth* is the mean average GDP growth rate over the period 2015-2019. *Start stringency* accounts for the number of days from 1 January 2020 without mandatory measures (e.g. 14 March 2020 = 74). *Delay* represents the period in days between starting mandatory measures (*Start stringency*) and the first registered COVID-19 infection. State fragility is measured by the Fund for Peace in 2018.

Source: Authors' own calculation.

take the sum of each country's ranking values for the stringency index and the number of deaths per one million inhabitants in the respective samples, with a lower number indicating better performance. Results (Table 5) confirm the baseline with the exception of the World Bank sample.

Finally, we run instrumental variable (IV) regressions explicitly accounting for some factors usually referred to in debates about government performance during the coronavirus crisis, namely political conditions as well as the speed with which governments responded to the pandemic. Accordingly, we use the fragile state index as measured by the Fund for Peace[3] and the time it took governments to respond as instruments for the CM index. We capture the latter using the number of days starting from 1 January 2020 without government-imposed stringency measures (e.g. 14 March 2020 = 74), and the number of days between the government applying initial social distancing measures and the occurrence of the first registered COVID-19 infection in the respective country. As shown in the last two columns of Table 1, there are substantial cross-country differences with regard to the speed of the government response. For example, while many countries started with some form of mandatory measures on social distancing even before the first case was recorded, others were more hesitant and waited until the first case was observed.

We run IV regressions for all specifications of the baseline. Results for the EIU sample countries of the first stage regression indicate that our instruments significantly explain cross-country differences in both indices (Table 6). Moreover, the second stage regression confirms the findings of the baseline. By contrast, for the other samples none of the chosen instruments is significant in the first stage regression, and the statistical significance of the CM index is only confirmed for the World Bank sample. Thus, the IV approach findings raise questions about the link between government performance and economic impact of the pandemic that should be addressed in the future when new data, either in the form of projections or as actual GDP growth, is available for a larger and more balanced set of countries.

## Conclusion

The COVID-19 pandemic has triggered the most severe global recession since World War II. However, as in the global financial crisis of 2008/2009, countries are affected differently. Based on the most recent OECD, IMF and World Bank projections of the GDP growth, this article presents evidence indicating that these differences are driven by the quality of the government response to the pandemic and by countries' exposure to the international transmission of the pandemic's impact, notably via tourism. Thus, governments performing well in handling the crisis can be expected to do better in terms of economic outcomes as well: good crisis management pays off.

We want to conclude by repeating that our analysis is based on growth projections only. Moreover, these projections involve country samples which are rather small and heterogeneous. Finally, a few robustness checks fail to support our main results. Thus, in several aspects these are preliminary results which we plan to continuously review and update as new evidence on 2020 GDP growth becomes available on a cross-country basis.

3 See https://fragilestatesindex.org/.
